# The impact of social media influencers’ bragging language styles on consumers’ attitudes toward luxury brands: The dual mediation of envy and trustworthiness

**DOI:** 10.3389/fpsyg.2022.1113655

**Published:** 2023-01-19

**Authors:** Wenting Feng, Dihui Chang, Hongjie Sun

**Affiliations:** Hainan University, Haikou, China

**Keywords:** social media, influencers, bragging language style, straightforward bragging, humblebragging, brand attitudes

## Abstract

On social media, luxury brand managers often use influencers’ bragging language as a marketing tool. As modesty is considered a virtue in the Chinese context, Chinese influencers tend to adopt a humblebragging language style. Research has examined the impact of bragging language styles on luxury brands and has found that humblebragging, which appears to be modest, has a negative influence on brand attitudes. From the perspective of social comparison theory, we proposed a dual mediation model of malicious envy and trustworthiness to reveal the internal mechanisms and moderating factors of the negative effects of humblebragging. The results of three experiments indicated that compared with straightforward bragging, humblebragging was more likely to elicit malicious envy and lower levels of trust in an influencer, resulting in negative attitudes toward the luxury brand endorsed. Moreover, this negative effect was stronger when the influencer lacked expertise or had high similarity with consumers. Our findings enrich the antecedents of social media influencer marketing and provide managers with implications for maximizing the effectiveness of influencer marketing by matching influencers with word-of-mouth content.

## 1. Introduction

Social media influencers have become an important channel for word-of-mouth (WOM) marketing. Influencer marketing worldwide is expected to reach $15 billion in 2022, and over 60% of consumers report that their purchase decisions are affected by influencers’ recommendations (FORBES, 2021). Especially for luxury brands, influencers’ WOM can highlight the symbolic social status of the brands in a vivid and interactive way. For example, luxury hotel brands such as Marriott, Bulgari, and Hilton have hired influencers on social media sites, such as TikTok and Little Red Book, to endorse the brands and present the high-end and luxurious experiences they offer by posting photos and live streaming (Zhang et al., 2021). Noticeably, influencer marketing of luxury brands often relies on the conspicuous display of travel experiences and highlights social symbolism. Studies have shown that such bragging can trigger consumers’ admiration and benign envy, thereby boosting brand attitudes and purchase intentions (Lee and Eastin, 2020). Thus, influencers’ bragging WOM has been widely found to be an effective marketing strategy for luxury brands (Chae, 2018; Jin and Muqaddam, 2019; Lee and Eastin, 2020).

Although bragging WOM can highlight the social symbolism of luxury brands, some studies have shown that straightforward bragging can be interpreted by social media users as boasting and showing off. Consumers may have negative perceptions of straightforward bragging, which directly presents the luxuriousness of brands (e.g., “Enjoying the private swimming pool exclusive to VIP guests and feeling like a winner in life”; Scopelliti et al., 2015). Humblebragging conveys the luxuriousness of brands through complaining (e.g., “As I cannot swim, the private swimming pool exclusive to VIP guests is not attractive”) and has been found to be a more effective strategy (Grant et al., 2018; Paramita and Septianto, 2021). Scholars have suggested that humblebragging can help conceal the boasting intention of WOM with humor and can enhance consumers’ affinity for celebrity endorsers (Wittels, 2012; Paramita and Septianto, 2021). Moreover, the relative effectiveness of humblebragging has been supported by research on celebrity endorsements (Grant et al., 2018; Paramita and Septianto, 2021). Furthermore, many managers believe that the merits of humblebragging may be even stronger in the Chinese market, as humility is widely considered a virtue in Chinese culture (Chen et al., 2017). Humblebragging thus seems more in line with moral norms and can weaken the negative connotation of showing-off behavior. Accordingly, an increasing number of influencers have adopted humblebragging, which is commonly known as “Versailles Literature” in China (e.g., “My boyfriend gave me a pink Lamborghini. This color is too rustic. How can I let him know that I do not like the color?”) instead of straightforward bragging language (Ren and Guo, 2021). In this way, social media influencers aim to alleviate consumers’ potential negative reactions to bragging to enhance their image and those of the brands they promote.

However, this raises the question of whether humblebragging is more effective than straightforward bragging. Although previous studies have emphasized the silver lining of humblebragging in celebrity endorsements, we argue that influencers’ humblebragging can negatively affect brand attitudes. Compared with celebrities, social media influencers are typically ordinary people with high similarity to their audience, and consumers are more likely to consider them social interaction targets and actively evaluate the social motive behind their WOM. For example, some consumers may think that an influencer who humblebrags is pretending to be modest or fails to cherish the travel experience. Therefore, humblebragging is likely to induce social comparison between consumers and influencers. Furthermore, on social media, where the social motivation is prominent, influencers’ bragging behavior can affect consumers’ cognition and emotion toward the influencer. On a cognitive level, when reading influencers’ humblebrags, which often conceal a boast with a complaint, consumers may think that the influencers are pretending to be modest and that they lack sincerity, thus decreasing consumers’ trust in them. On an emotional level, humblebragging can cause consumers to perceive a low level of deservingness, triggering negative social emotions such as envy and disgust. Such negative cognitive (i.e., low trustworthiness) and emotional (i.e., high malicious envy) evaluations of influencers may spill over to the luxury brands that the influencers endorsed, resulting in consumers having negative brand attitudes. Thus, hiring influencers to share luxury brands in a humblebragging style may backfire and diminish brand attitudes.

This research investigated the negative effect of influencers’ humblebragging on the luxury brands they endorse as well as its internal mechanism and moderating factors. Studies on bragging WOM have emphasized the positive effects of humblebragging in the context of celebrity endorsements (Grant et al., 2018; Paramita and Septianto, 2021). However, we argue that humblebragging can have negative effects when used by social media influencers. Moreover, although previous influencer studies have mainly adopted the information communication perspective (Breves et al., 2019; Lou and Yuan, 2019), we drew inferences from social comparison theory to propose a dual mediation model to explain the mechanism underlying consumers’ attitudes toward the brands endorsed by influencers. We suggest that the envy and lack of trust aroused by consumers’ social comparisons with influencers can spillover to the endorsed brands and hinder the effectiveness of WOM marketing. This research enriches the understanding of the antecedents to influencer marketing effectiveness and provides practical implications for brand managers regarding how to maximize the effectiveness of influencer marketing through personalized WOM content.

## 2. Literature review

### 2.1. Influencer marketing

Social media influencers are users who accumulate a large number of followers in specific fields and endorse brands on social media platforms (De Veirman et al., 2016). Influencers differ from celebrity endorsers in three distinct ways. First, they are typically ordinary people, and thus they typically have a high degree of similarity with consumers (Jin, 2018). Second, consumers generally consider the WOM shared by influencers more authentic than celebrity endorsements (Audrezet et al., 2020; Lee and Eastin, 2021). Third, influencers are typically experts who have built their influence in a specific domain (Jin et al., 2019; Ki and Kim, 2019). The similarities and differences between social media influencers and celebrity endorsers are summarized in Table 1.

**Table 1:** The similarities and differences between influencer and celebrity endorsements.

	Social media influencer	Celebrity endorsers
Influence	High, based on the number of social media followers	High, based on reputation outside social media
Similarity	High	Low
Authenticity	High, personalized endorsement content	Low, standardized endorsement content
Interactivity	High (like, comment, forward)	Low
Brand endorsements	Less obvious	More obvious

Social media influencers present themselves through posts on social media and can potentially affect consumers’ attitudes and purchase decisions (Appel et al., 2020). Influencers have a high degree of similarity with consumers, which can effectively reduce consumers’ psychological resistance and improve their preference for advertising (Breves et al., 2019; Ki and Kim, 2019). Influencers can also effectively improve consumers’ product attitudes (Torres et al., 2019) and trigger positive emotions (Trivedi et al., 2021). For example, Torres et al. (2019) found that popular influencers can significantly improve product attitudes. Finally, influencers can improve consumers’ purchase intentions for the endorsed brands (Jin and Ryu, 2020; Ki et al., 2020). Ki et al. (2020) pointed out that when consumers have positive emotions toward a social media influencer, those positive emotions are transferred to the products, thus enhancing purchase intentions.

Given the extensive impact of social media influencers on consumer attitudes and behaviors, research has explored the following antecedents of influencer marketing effectiveness: (1) individual influencer characteristics, including influence (Kay et al., 2020), attractiveness (Lou and Yuan, 2019), expertise (Ki and Kim, 2019; Ki et al., 2020; Chen et al., 2021), and credibility (Jin et al., 2019; Lee and Eastin, 2020); (2) endorsement content characteristics, such as vividness (Jin and Ryu, 2020; Lou and Yuan, 2019), interactivity (Ki and Kim, 2019), and endorsement display form (Stubb and Colliander, 2019; Kim and Kim, 2021); (3) the relationship between an influencer and the endorsed brand, such that the better an influencer’s expertise fits the category of the endorsed product, the more positive consumers’ attitudes toward the product and the higher their purchase intentions (Breves et al., 2019; De Cicco et al., 2021); and (4) the similarity between influencers and consumers. Some research has adopted the self-congruency perspective and found that similarity between consumers and influencers improves influencer credibility and thus brings about positive brand attitudes (Ki et al., 2020; Yuan and Lou, 2020). Table 2 summarizes the independent variables, dependent variables, and mediating mechanisms in the literature on influencer marketing.

**Table 2:** Recent research on influencer marketing.

Independent category	Independent	Mediators	Dependent	Citations
Individual characteristics	Expertise	Brand attitude	Purchase intention	Hughes et al. (2019)
	Attractiveness	Brand attitude	Purchase intention	Torres et al. (2019)
	Attractiveness	Message credibility	Purchase intention	Lou and Yuan (2019)
	Influence	Influencer likeability	Purchase intention	Kay et al. (2020)
	Credibility	Brand image	Purchase intention	Jin and Ryu (2020)
	Similarity	Brand attachment	Purchase intention	Ki et al. (2020)
	Similarity	Psychological resistance	Attitude toward the ad	Breves et al. (2019)
Endorsement styles and characteristics	Content value	Message credibility	Purchase intention	Ki and Kim (2019)
	Content valence	Message credibility	Purchase intention	Balaji et al. (2021)
	Interactivity	Motivation of mimic	Purchase intention	Ki and Kim (2019)
	Entertainment	Engagement	Purchase intention	Magno (2017)
	Linguistic style	Psychological resistance	Attitude toward the ad	Lee and Theokary (2020)
	Endorsement nature	Advertising identity	Product attitude	Kim and Kim (2021)
	Endorsement nature	Influencer credibility	Attitude toward the ads	De Cicco et al. (2021)
Relationship with endorsed brands	Brand-endorser fit	Message credibility	Purchase intention	Breves et al. (2019)
	Product-endorser fit	Affective motive	Product attitude	Kim and Kim (2021)
	Product-endorser fit	Message credibility	Attitude toward the ads	Belanche et al. (2021)
Relationship with consumers	Self-congruency	Influencer credibility	Product attitude	Yuan and Lou (2020)
	Self-congruency	Engagement	Purchase intention	Sánchez-Fernández and Jiménez-Castillo (2021)
	Self-disclosure	Para-social interaction	Normative commitment	Wang and Hu (2022)

Regarding the mechanism of influencer marketing, the literature has drawn insights from the information communication perspective and identified consumers’ trustworthiness as an important mediating factor. Some scholars have found that influencers can improve purchase intentions because consumers consider influencers reliable information sources (Lou and Yuan, 2019; Yuan and Lou, 2020). For example, Jin and Ryu, (2020) found that compared with traditional celebrity endorsements, consumers are more likely to trust the products recommended by influencers. Lee and Eastin (2021) showed that when consumers regard influencers as reliable information sources, they are less likely to feel that the influencers are “hard selling” and are more receptive to their WOM.

In summary, studies have mainly focused on the effects of influencers’ individual characteristics (e.g., similarity and expertise), emphasizing the positive effects of these individual characteristics on brand attitudes. Relatively few studies have discussed the potential role of influencers’ WOM language styles. That is, research has focused on who the influencers are while largely ignoring how they post on social media. However, distinct from celebrity endorsements, influencer WOM is personalized and often an avenue of self-presentation (Shen, 2021). As influencers are motivated by self-image enhancement, they are more likely than celebrities to mention brands using humblebragging (Paramita and Septianto, 2021), which may induce social comparison between consumers and influencers, triggering complex social emotions. However, influencer WOM often has a personalized language style, which may have a distinct effect on consumers’ attitudes and behaviors. Therefore, this research explored the potential impacts, mechanism, and moderating factors of influencers’ language styles on consumers’ brand attitudes.

### 2.2. Bragging language style

On social media, consumers often seek self-enhancement through bragging about luxury travel experiences (Pelletier and Collier, 2018; Liu and Li, 2021; Cohen et al., 2022). For example, Liu and Li (2021) proposed that consumers seek “bragging rights” by posting their travel experiences on social media to build a favorable self-image and gain recognition from others. Such bragging is especially pervasive among social media influencers (Chae, 2018; Audrezet et al., 2020).

Moreover, influencers may adopt various bragging language styles (Paramita and Septianto, 2021). Research has proposed two categories of bragging language style: straightforward bragging and humblebragging (Sezer et al., 2018). Straightforward bragging refers to posts that directly emphasize an individual’s social status or desirable possessions, with obvious self-enhancement intention (Scopelliti et al., 2015). For example, when endorsing a luxury hotel, an influencer might post the following: “Since I’m a VIP customer at this five-star hotel, the manager frequently asks about my requests for my stay.” Such WOM presents the sender’s social status in a straightforward manner. In contrast, humblebragging refers to posts that use a complaint to conceal the individual’s self-enhancement intention. For example, when recommending a luxury hotel, an influencer might post the following: “I’m a VIP customer at this five-star hotel, and I find it annoying that the manager frequently disturbs me to ask if I have any requests.”

Regarding the effectiveness of the two bragging language styles, many studies have highlighted the negative effect of straightforward bragging. Scholars have found that straightforward bragging reveals an individual’s self-serving motivation (Berman et al., 2015), which often violates social norms such as modesty (Packard et al., 2016). As a result, scholars have suggested that straightforward bragging can elicit negative attitudes toward the influencer (Scopelliti et al., 2015) and the brands they promote (Ferraro et al., 2013). In contrast, researchers have found that humblebragging can be perceived as reflecting the influencer’s humility, which helps improve their self-image and gains recognition from others (Sezer et al., 2018). For example, Paramita and Septianto (2021) showed that when celebrities used humblebragging (vs. straightforward bragging) to recommend brands on social media, consumers favorable brand attitudes were enhanced.

Moreover, a few recent studies have explored potential moderating factors (Doty, 2019; Chen et al., 2020). Chen et al. (2020) investigated consumers’ bragging behavior in an online review platform and found that the marketing effects of straightforward bragging and humblebragging depend on the influencer’s relative expertise. They found that when consumers have a lower level of expertise than the influencer, straightforward bragging is more effective than humblebragging in improving brand attitudes. Doty (2019) studied bragging behaviors in the context of social media and found that gender moderates the relative effectiveness of the two bragging language styles. These studies have demonstrated the moderating effect of individual characteristics on the effectiveness of bragging language style. Table 3 summarizes the independent variables, dependent variables, mediators, and moderators in the recent studies on bragging language style.

**Table 3:** Review in the recent studies on bragging language style.

Independent	Mediators	Moderators	Dependent	Citations
Bragging vs. humblebragging	Reviewer likability, benign envy	Reviewer expertise	Brand evaluation	Chen et al. (2020)
Straightforward brag vs. humblebrag	/	Gender of braggart	Admiration, liking	Doty (2019)
Straightforward bragging vs. humblebragging	Amusement, irritation	Celebrity vs. influencer endorsement	Brand attitude	Paramita and Septianto (2021)
Straightforward bragging	Motive perceptions, expertise perceptions	Trust cues	Persuasion	Packard et al. (2016)
Straightforward bragging vs. humblebragging vs. complaining	Perceived sincerity	/	Liking	Sezer et al. (2018)

Nevertheless, there are two limitations in the literature on bragging language styles. Studies have largely focused on online review platforms where the users are anonymous. There remains a lack of insight into the potential role of bragging in the context of social media influencer marketing. As social media influencers have frequent interactions and established relationships with consumers, their bragging behavior can easily trigger social comparison by consumers and social emotions such as envy. Furthermore, to the best of our knowledge, there is little in-depth research on the internal psychological mechanism of bragging language style. Although some scholars have shed light on the mediating effect of consumers’ perceptions of influencers (Chen et al., 2020), understanding consumers’ response to influencers’ bragging merely from a cognitive perspective may be insufficient. As social emotion (e.g., envy) is more prominent on social media (Liu et al., 2019), this research sought to explain the mechanism of the relative effectiveness of the two bragging language styles by considering the mediating role of envy.

### 2.3. Envy on social media

Social comparison theory holds that in relatively subjective social evaluations, individuals tend to self-evaluate through comparisons with similar others, and this affects their thinking, emotions, and behaviors in the social context (Festinger, 1954). Individuals may make upward, downward, or parallel social comparisons according to their emotions or motivations (Johnson and Knobloch-Westerwick, 2014). In downward social comparison, people compare themselves with those they perceive to be inferior to them in terms of living environment, quality, or ability (Kim, 2022). Conversely, in upward social comparison, people compare themselves with those whose attributes and abilities they perceive as superior to their own (Dinh and Lee, 2022). Envy is an emotional consequences of upward social comparison (Smith, 2000).

Envy is an emotion based on social comparison, and it refers to the negative emotional experience that occurs when individuals realize that others have advantages and achievements that they desire (Lange and Crusius, 2015). In the tourism context, consumers may be envious when they see that others have superior experiences (for example, staying in a luxury hotel; Feng et al., 2021a,b). In the context of influencer marketing, because influencers seek to enhance their ability to influence others through favorable self-presentation, they are more likely to trigger consumers’ envy (Chae, 2018; Chen et al., 2020).

Social psychology research has categorized two types of envy: benign and malicious (Van de Ven et al., 2009). Benign envy refers to the emotional experience of envy that involves admiration. Individuals with strong motivation for self-improvement (Van de Ven et al., 2009) tend to reduce the gap with a social comparison target by trying to improve themselves (Lange and Crusius, 2015; Salerno et al., 2019). Malicious envy is an emotional experience of envy that involves hostility. Individuals are motivated to devalue the superiority of a social comparison target (Van de Ven et al., 2009, 2011a), often through aggressive strategies such as schadenfreude (Van de Ven and Zeelenberg, 2015; Lange et al., 2016). On social media, whether consumers experience benign or malicious envy depends on the extent to which they consider a social comparison target superior, i.e., the extent to which they consider the target to have achieved superiority through personal effort (Feng et al., 2021a,b). For example, individuals do not attribute the wealth of those born to wealthy families to their personal efforts, which is likely to lead to malicious envy. In other words, when individuals consider a social comparison target to have little deservingness of their superiority, they are more inclined to feel malicious envy (Van de Ven et al., 2012; Lange et al., 2016).

Envy can have a profound effect on consumer attitudes and behaviors. In the context of product consumption, benign envy improves consumers’ purchase intentions for products and services, whereas malicious envy may diminish purchase intentions (Hajli et al., 2018; Van de Ven et al., 2011b). Lin (2018) found that consumers experiencing benign envy are inclined to choose the brand owned by the target of their envy, whereas those feeling malicious envy tend to choose competitive brands. In the tourism context, when consumers see their friends sharing travel experiences on social media, benign envy leads to a positive attitude toward the destination visited (Hajli et al., 2018; Liu et al., 2019), whereas malicious envy may elicit a negative attitude toward the destination (Feng et al., 2021a,b). Table 4 summarizes the independent variables, dependent variables, mediators, and moderators in recent research on envy.

**Table 4:** Recent research on envy.

Independent	Mediators	Moderators	Dependent	Citations
Bragging type	Benign envy	Reviewer expertise	Brand evaluation	Chen et al. (2020)
Social comparison, self-presentation	Travel envy	/	Visit intention	Hajli et al. (2018)
Perceived similarity with reviewer writer	Malicious envy	Perceived deservingness of reviewer writer	Visit intention	Feng et al. (2021a)
Being envied perception	Pride, Anxiety	Social tie strength	Self-brand connection	Feng et al. (2021b)
Psychological entitlement	Malicious envy	Type of testimonial endorser	Attitude toward the destination	Martin et al. (2019)
Positive travel experience	Benign envy	Similarity, trait self-esteem	Destination visit intention	Liu et al. (2019)
Material post content	Perceived intention of showing off	/	Envy type (benign vs. malicious) purchase intention	Lin (2018)
Social comparison, self-presentation	Travel envy, domestic travel behavior	/	Social return	Sharma et al. (2022)
Envy	Self-promotion	/	Sharing travel “selfies”	Taylor (2020)

## 3. Hypothesis development

### 3.1. The effects of straightforward bragging and humblebragging on brand attitudes

On the basis of social comparison theory, we propose that straightforward bragging and humblebragging produce different self-discrepancy perceptions that affect consumers’ attitudes toward a brand endorsed by influencers. Specifically, we argue that compared with straightforward bragging, influencers’ humblebragging may result in negative brand attitudes. The social media literature has shown that individuals’ social comparison with other social media users leads to self-discrepancy perceptions and affects their evaluations of those users (Aw and Chuah, 2021). We argue that compared with straightforward bragging, humblebragging is more likely to induce upward social comparison between consumers and influencers. Such upward social comparison may trigger negative perceptions and emotions about the influencers. As social comparison is likely to induce envy (Appel et al., 2016), we propose that humblebragging is likely to trigger malicious envy. For example, when an influencer shares WOM about luxury brands with a complaint (e.g., “The hotel has a luxurious swimming pool, but it’s useless to me because I cannot swim”), consumers may consider the influencer undeserving of the luxury brand, leading to negative emotions. As individuals who feel malicious envy are motivated to alleviate their self-discrepancy by devaluing the social comparison target (Feng et al., 2021a,b), they may have a negative attitude toward influencers who humblebrag (Sezer et al., 2018). Finally, as consumers’ evaluations and perceptions of influencers can easily spill over to the endorsed brand (Belanche et al., 2021), we propose that influencers’ humblebragging (vs. straightforward bragging) may prompt consumers to negatively evaluate the luxury brands the influencers recommend. Hence, we propose the following:

*H1*: Compared with straightforward bragging, influencers’ humblebragging may diminish consumers’ brand attitudes toward luxury brands.

### 3.2. The mediating effects of malicious envy and trustworthiness

Most of the relevant literature has considered trustworthiness the key to explaining the mechanism of bragging language style from the cognitive perspective (Chen et al., 2020). However, in the social media context, influencers’ bragging is likely to trigger upward social comparison by consumers, which may elicit malicious envy (Smith, 2000). Therefore, integrating the social emotion perspective, we propose that the negative effect of the humblebragging language style is driven by the dual mediating mechanisms of malicious envy and trustworthiness. Studies have found that envy can explain the positive effect of influencers on purchase intentions (Jin et al., 2019; Jin and Ryu, 2020). For example, Jin and Ryu (2020) found that consumers are likely to feel benign envy after browsing influencers’ selfies and that they are willing to buy the products used by the influencers. Chen et al. (2020) also found that benign envy is the mechanism underlying the positive effect of bragging WOM. Whereas previous studies have mainly discussed the role of benign envy, we propose that malicious envy can explain the negative effects of humblebragging. Specifically, compared with straightforward bragging (e.g., “The hotel provides luxurious shuttle services to VIP clients, and picks me up from the airport with a luxury sports car”), when an influencer uses humblebragging (e.g., “The hotel picked me up from the airport with a luxury sports car, yet I felt uncomfortable”), consumers may think that the influencer did not cherish or deserve the service experience. Such perceptions of low deservingness may trigger consumers’ malicious envy toward the influencer (Van de Ven et al., 2009; Lange and Crusius, 2015).

However, a majority of the relevant studies have identified trust as a key antecedent of the effectiveness of influencers’ WOM (Jin et al., 2019; Lou and Yuan, 2019). For example, Lou and Yuan (2019) found that consumers with high trust in an influencer showed increased purchase intentions for the brands that influencer recommended. This research extends the literature to the service context and proposes that humblebragging may reduce the perceptions of an influencer’s trustworthiness. Social psychology studies have found that when individuals humblebrag, their audience may perceive them as having a low level of sincerity (Sezer et al., 2018). Hence, we argue that when an influencer endorses luxury brands *via* humblebragging, consumers may perceive the influencer as pretending to be modest and their WOM as insincere; as a result, they are likely to consider the influencer an unreliable information source. Consumers’ perceptions of an influencer’s low trustworthiness can negatively affect their attitudes toward the brands recommended by the influencer (Jin and Ryn 2020; Kim and Kim, 2021). Thus, we propose the following:

*H2*: Consumers’ malicious envy and perceptions of social media influencers’ trustworthiness are parallel mediators in the effects of bragging language styles on brand attitudes.

### 3.3. The moderating effects of influencers’ individual characteristics

We propose that influencers’ individual characteristics may moderate the relative effectiveness of both types of bragging. Compared with celebrity endorsements, influencers often have high similarity to consumers and expertise in a certain field (Gretzel, 2018). Research has shown that similarity and expertise are two important variables that characterize social media influencers (Breves et al., 2019; Ki and Kim, 2019; Ki et al., 2020). Moreover, when selecting influencers to endorse a product, managers often consider the influencers’ expertise in the industry and their similarity to the target consumer segment. Therefore, we propose influencers’ similarity and expertise as two factors that moderate the effects of bragging language styles on brand attitudes.

#### 3.3.1. Consumers’ perceived similarity to influencers

We suggest that consumers’ perceived similarity to influencers may aggravate the negative effect of humblebragging. On social media, consumers tend to make social comparisons with individuals they perceive as similar to themselves (Lin, 2018). The prominence of upward social comparison has been identified as a key factor that triggers malicious envy among consumers (Appel et al., 2016). In the context of influencer marketing, as consumers have a strong affinity for influencers (Paramita and Septianto, 2021), we argue that consumers are likely to consider influencers social comparison targets. During social comparison, consumers may evaluate the target’s deservingness of their superiority (Lin, 2018; Feng et al., 2021a,b). Furthermore, because humblebragging reveals an individual’s superiority *via* a complaint, consumers tend to perceive a low level of deservingness. For example, when an influencer complains about their stay in a luxury hotel, consumers may think that the influencer did not deserve the luxurious experience, and thus they experience malicious envy. Finally, because upward social comparison may result in a low evaluation of the social comparison target (Smith, 2000), we argue that the social comparison induced by humblebragging may encourage consumers to evaluate influencers as having low trustworthiness. For example, consumers may think that an influencer who humblebrags lacks sincerity, and hence, they are not trustworthy. In contrast, when consumers perceive influencers as having low similarity, they are less likely to make a social comparison (Feng et al., 2021a,b). As a result, consumers may show low levels of malicious envy and trustworthiness, resulting in a favorable attitude toward the brands the influencer promotes. Hence, we propose the following:

*H3*: When consumers perceive social media influencers as being similar to themselves, they may experience strong feelings of malicious envy and perceive influencers who humblebrag as untrustworthy.

#### 3.3.2. The moderating effect of influencers’ expertise

**Figure 1:**
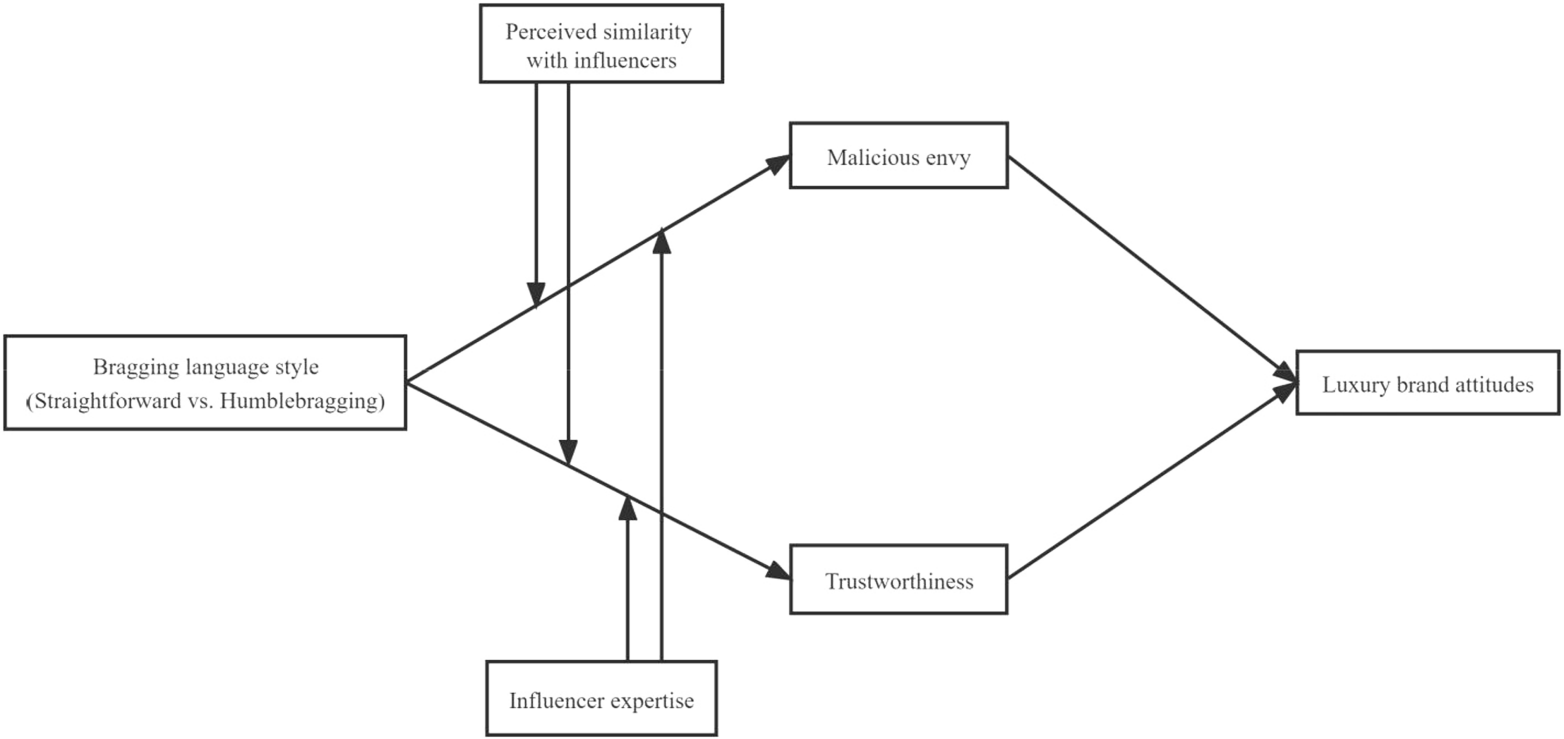
Conceptual model.

We propose that influencers’ expertise in the products they endorse may attenuate the effects of bragging language style. First, we argue that influencers’ expertise affects consumers’ tendency for social comparison. When interacting on social media (Gligor and Bozkurt, 2021; Wang, 2021), individuals may speculate about the social motivation underlying other users’ WOM (Berger, 2014). When interacting with users with low levels of expertise, consumers tend to attribute those users’ WOM to social motivations such as self-enhancement, which induces social comparison (De Angelis et al., 2012). Drawing inferences from these findings, we propose that influencers with low expertise are more likely to trigger social comparison and thereby malicious envy. For example, when an influencer in the fitness field recommends a luxury hotel *via* humblebragging (e.g., “The hotel offers welcome snacks to VIP guests exclusively, but I could not enjoy them because I need to stay fit”), consumers are likely to make a social comparison and experience malicious envy. In contrast, when influencers have a high level of expertise, consumers are more inclined to attribute their motivation to post about luxury possessions or experiences to information sharing. For example, when a travel expert recommends a hotel brand by humblebragging, consumers are more inclined to consider the WOM travel-related information. Furthermore, expertise affects consumers’ perceptions of trustworthiness (Packard et al., 2016). Studies have found that consumers attribute the WOM of influencers with high expertise to non-social motives, such as information sharing, and perceive the influencers as a reliable source of information (Pop et al., 2022). In this case, the negative effects of humblebragging are weakened when influencers have expertise in the luxury brands they recommend. In contrast, when influencers without expertise adopt humblebragging, consumers may attribute their WOM to a self-enhancement motive and evaluate them as untrustworthy (Figure 1). As such, we propose the following:

*H4*: When influencers lack expertise, consumers experience malicious envy and perceive influencers who humblebrag as untrustworthy.

The conceptual model is illustrated in Figure 1.

We conducted three experiments to test our hypotheses. Studies 1A and 1B examined the main effects of straightforward bragging and humblebragging on luxury brand attitudes and the mediating effects of malicious envy and perceived trustworthiness of influencers (H1 and H2). Studies 2 and 3 tested the moderating effects of perceived similarity with the influencer (H3) and influencer expertise (H4), respectively.

## 4. Study 1: The influence of bragging language style on brand attitudes

### 4.1. Study 1A

#### 4.1.1. Purpose and participants

Study 1A tested the main effect and underlying mechanism of bragging language style on luxury brand attitudes. It involved a between-subjects experiment with two groups: straightforward bragging and humblebragging. We recruited 202 participants from SoJump.com, a professional Chinese survey platform, and randomly assigned the participants to one of the two groups. Among the participants, 40.6% were men, 31.7% were between 26 and 30 years old, 74.8% were enterprise employees, and 38.1% had an annual income of RMB60,000 to RMB100,000.

#### 4.1.2. Design and procedures

The study procedure was as follows. First, the participants were asked to browse a WOM post by an influencer on the social media site Little Red Book. The influencer named “Lee” shared his recent stay at a luxury Peninsula Hotel in Paris. We chose luxury hotel brands as the stimuli because influencers’ WOM regarding luxury hotels are pervasive on social media, occurring even more frequently than WOM about material possessions. Following Chen et al. (2020), we manipulated bragging language style through online WOM content. In the straightforward bragging scenario, the WOM directly highlighted the influencer’s superiority: “When I entered the room, there was complimentary champagne and a chocolate on the table. The champagne and chocolate were exclusive to VIP guests like me… In the evening, I went to the Michelin-starred restaurant downstairs. The restaurant manager recognized me and asked me several times how the food was during the meal.” In the humblebrag scenario, the WOM content emphasized the influencer’s superiority *via* complaints: “When I entered the room, there was champagne and chocolate on the table. I am trying to lose weight, so I dared not eat these! … The restaurant manager recognized me and asked me many times how the food was during the meal. I felt a little annoyed since I was frequently disturbed.” The scenarios are shown in Supplementry Appendix 1.

After reading the influencer’s WOM, the participants were asked to evaluate their perceptions of the bragging language styles, their feelings of malicious envy, the trustworthiness of the influencer, and their brand attitude toward the Peninsula Hotel. Bragging language style was measured using the item “I think his bragging is direct/contains a little complaint,” which was adopted from Sezer et al. (2018). Malicious envy was assessed using four items adapted from Lange et al. (2018), e.g., “I think he is enviable” and “I have some negative views of him” (Cronbach’s *a =* 0.772). Trustworthiness was measured using four items adapted from Lou and Yuan (2019), such as “He is sincere” and “He is honest” (Cronbach’s *a =* 0.849). Brand attitude was assessed with three items adapted from Lee et al. (2017), including “I like the Peninsula Hotel,” “I think the Peninsula Hotel is a good hotel,” and “I have a negative view of the Peninsula Hotel” (reversed; Cronbach’s *a =* 0.760). These questions all used a 7-point Likert scale (1 “strongly disagree”; 7 “strongly agree”). The scales are provided in Supplementry Appendix 2.

#### 4.1.3. Results

*Manipulation Check.* The results of the independent sample t-test showed significant differences in the participants’ perceptions of language style between the two groups. Specifically, compared with the humblebragging condition (*M*_humblebragging_ = 3.55, SD = 1.25), the participants in the straightforward bragging condition were more inclined to consider the influencer’s bragging as direct (*M*_straightforward bragging_ = 4.88, SD = 1.09, *p* < 0.001). Compared with the straightforward bragging condition (*M*_straightforward bragging_ = 2.54, SD = 1.30), those in the humblebragging condition thought that the influencer’s bragging contained a little complaint (*M*_humblebragging_ = 3.52, SD = 1.61, *p* < 0.001).

**Figure 2:**
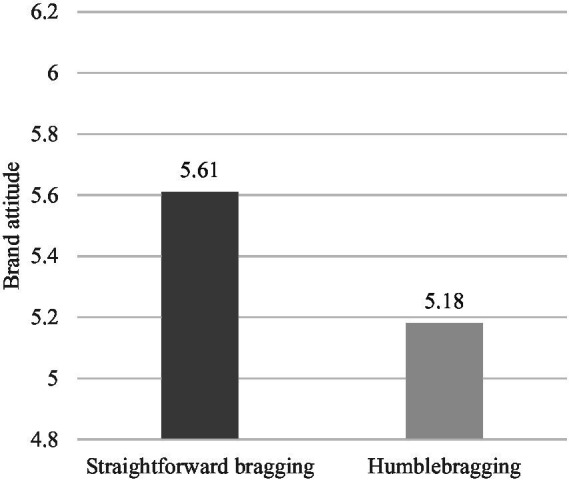
Study 1A: The impact of bragging language style on brand attitudes.

*Brand Attitude.* The results of the one-way analysis of variance (ANOVA) indicated significantly different luxury brand attitudes [*F*(1,200) = 10.790, *p* = 0.001] between the two groups. As shown in Figure 2, the participants in the humblebragging condition (*M*_humblebragging_ = 5.18, SD = 1.00) showed significantly lower brand attitude than those in the straightforward bragging condition (*M*_straightforward bragging_ = 5.61, SD = 0.83), supporting H1.

*Mediation Analysis.* We used Hayes’s (2017) PROCESS method (Model 4, Bootstrap sample = 5,000) to test the dual mediating effects of malicious envy and trustworthiness. The direct effect of bragging language style on brand attitude was not significant (*β* = 0.058, 95% CI: [−0.199, 0.315]). The indirect effects of malicious envy (*β* = −0.125, 95% CI: [−0.245, −0.027]) and trustworthiness (*β* = −0.359, 95% CI: [−0.510, −0.226]) were both significant. The results indicated that malicious envy and influencer trustworthiness had parallel mediating effects in the negative role of humblebragging language style.

### 4.2. Study 1B

#### 4.2.1. Purpose and participants

The stimulus of Study 1B was integrated into the Versailles Language style of Chinese tourism influencers to re-examine the main effect of WOM bragging language style. Study 1B was a between-subjects experiment with two groups: straightforward bragging and humblebragging. We recruited 110 participants from the professional questionnaire platform Credamo.com, and randomly assigned them to one of the two groups. Among the participants in Study 1B, 34.5% were men, 35.5% were between 19 and 25 years old, 44.5% were private employees, and 37.3% had stayed in a luxury hotel once or twice. In addition, 31.8% of the participants spent 1–5 h per week on Weibo and 31.8% spent 6–10 h.

#### 4.2.2. Design and procedures

First, the participants were asked to imagine that they were browsing influencers’ posts on the social media platform Weibo and that they saw travel notes and pictures shared by travel influencer “Xiao Ke” about her recent stay in the luxury Banyan Tree Hotel. In the experimental condition, we referred to the Versailles Language style elements of domestic tourism influencers and manipulated the bragging language style *via* WOM content. Specifically, in the straightforward bragging condition, the WOM was as follows: “The vast river view, the huge room, how happy I was to live in it! … the manager sent me a white gift box printed with ‘Dessert Noble - French Raspberry macaron.’ The gift box was very nice and looked very stylish!” In the humblebragging condition, the WOM was “The vast river view, the huge room, how lonely it was to live in! … the manager sent me a white gift box printed with ‘Dessert Noble - French Raspberry macaron.’ The gift box was crude. How can I let him know I do not like the design?”

After reading the posts, the same scales as in Study 1A were used to measure the participants’ perceptions of bragging language style, malicious envy (Cronbach’s *a =* 0.762), influencer trustworthiness (Cronbach’s *a =* 0.930), and brand attitude toward the Banyan Tree Hotel (Cronbach’s *a* = 0.851). To eliminate the potential confounding effects of social comparison, we followed Gibbons et al. (1999) and measured it with three items (Cronbach’s *a* = 0.920), such as “I would compare myself to this influencer.” These questions were also assessed on a 7-point Likert scale (1 “strongly disagree”; 7 “strongly agree”).

#### 4.2.3. Results

*Manipulation Check.* The results of the one-way ANOVA showed that compared with the humblebragging condition (*M*_humblebragging_ = 4.67, SD = 1.43), the participants in the straightforward bragging condition were more inclined to think that the influencer’s bragging was direct (*M*_straightforward bragging_ = 5.36, SD = 1.22, *p =* 0.007). Compared with the straightforward bragging condition (*M*_straightforward bragging_ = 2.09, SD = 1.04), the participants in the humblebragging condition were more likely to think that the influencer’s bragging contained a little complaint (*M*_humblebragging_ = 4.58, SD = 1.42, *p* < 0.001).

**Figure 3:**
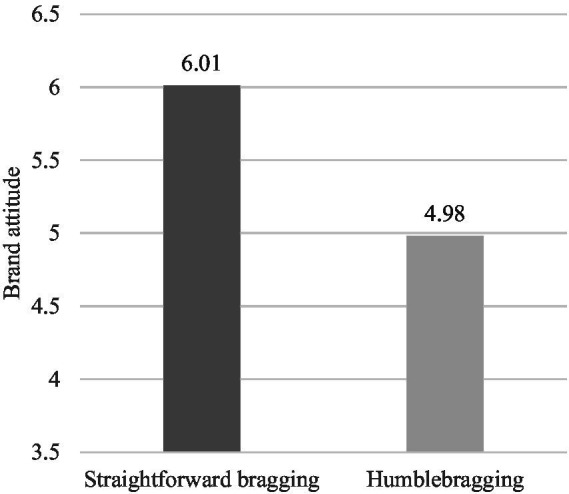
Study 1B: The impact of bragging language style on brand attitudes.

*Brand Attitude.* We conducted a one-way ANOVA on brand attitude. The results indicated a significant difference between the two groups’ brand attitudes toward the Banyan Tree Hotel (*F*(1,108) = 37.003, *p* < 0.001). Then, we repeated the ANOVA with social comparison tendency as a control variable, and the results were similar [*F*(1,107) = 41.501, *p* < 0.001, η^2^ = 0.279], indicating that social comparison did not influence the effect of bragging language style on brand attitude. Specifically, as shown in Figure 3, the participants in the humblebragging condition (*M*_humblebragging_ = 4.98, SD = 1.10) reported significantly lower brand attitude than those in the straightforward bragging condition (*M*_straightforward bragging_ = 6.01, SD = 0.59). These results revalidated H1.

*Mediation Analysis.* Finally, we used Hayes’s (2017) PROCESS method (Model 4, Bootstrap sample = 5,000) to check the dual mediating effects of malicious envy and trustworthiness. The direct effect of bragging language style on brand attitude was not significant (*β* = −0.075, 95% CI: [−0.378, 0.229]). The indirect effects of malicious envy (*β* = −0.462, 95% CI: [−0.782, −0.183]) and trustworthiness (*β* = −0.488, 95% CI: [−0.731, −0.284]) were significant, indicating that malicious envy and influencer trustworthiness had parallel mediating effects in the negative role of humblebragging language style. These results supported H2.

## 5. Study 2: The moderating effect of influencer similarity

### 5.1. Purpose and participants

Study 2 aimed to examine the moderating effect of perceived similarity with influencers. Study 2 used a 2 (bragging type: straightforward bragging vs. humblebragging) × 2 (similarity: high vs. low) between-subjects design. We recruited 202 participants from a university in China and randomly assigned them to one of the four experimental conditions. Of the participants, 43.1% were men, 98.5% were between 19 and 25 years old, 69.8% traveled once or twice a year, and 51.5% stayed in luxury hotels once or twice a year.

### 5.2. Design and procedures

#### 5.2.1. Procedure

The participants were asked to imagine themselves browsing travel notes and pictures shared by influencers on the travel social networking site mafengwo.com and that they saw the travel influencer “Dou Dou” share his experience at the Marriott Resort hotel in Sanya. The manipulation of influencers’ bragging language style was similar to that in Study 1, and the details of the scenario are shown in Supplementry Appendix 1. Following the study of Lin (2018), we manipulated perceived similarity through the demographic information of the influencer, including age and occupation. In the high similarity scenario, the influencer was described as an ordinary college student who was just 20 years old. In the low similarity scenario, the influencer was described as a middle-aged man in his 50s who worked as an executive at a well-known foreign company.

#### 5.2.2. Measures

We measured the participants’ perceptions of bragging language style, malicious envy (Cronbach’s *a =* 0.760), influencer trustworthiness (Cronbach’s *a =* 0.876) and brand attitude (Cronbach’s *a =* 0.752) using the same items as in Study 1A. The participants’ perception of similarity with the influencer was measured using two items rated on a 7-point bipolar scale adapted from Lin (2018): “He is about the same age as me” and “He is similar to me” (Cronbach’s *a =* 0.633). Finally, we assessed the participants’ perceptions of the authenticity of the influencer’s WOM with the following item: “I think the influencer’s WOM is authentic.”

### 5.3. Results

#### 5.3.1. Manipulation check

Of the two groups, the participants in the humblebragging condition were more inclined to perceive that the WOM contained a complaint [*M*_humblebragging_ = 4.98, SD = 1.07; *M*_straightforward bragging_ = 3.04, SD = 1.08; *F*(1,200) = 165.458, *p* < 0.001]; whereas in the straightforward bragging condition, the participants were more inclined to think that the WOM used direct language [*M*_humblebragging_ = 3.33, SD = 1.16; *M*_straightforward bragging_ = 5.14, SD = 1.10; *F*(1,200) = 131.065, *p* < 0.001]. In the manipulation test of influencer similarity, compared with the participants in the low similarity condition, the participants in the high similarity condition were more likely to perceive the influencer as similar to themselves [*M*_high similarity_ = 4.72, SD = 1.11; *M*_low similarity_ = 2.52, SD = 1.51; *F*(1,200) = 136.43, *p* < 0.001].

#### 5.3.2. Brand attitude

**Figure 4:**
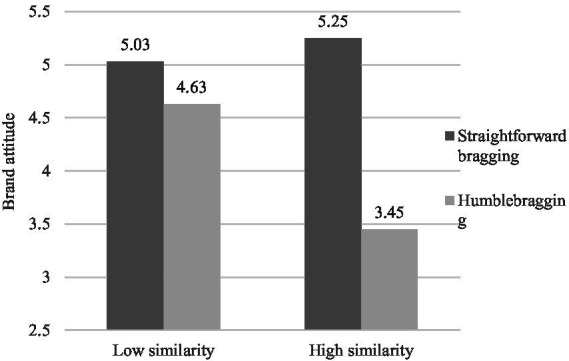
Study 2: The effect of bragging style and similarity on brand attitude.

We conducted a two-way ANOVA on brand attitude with perceived authenticity of WOM as a control variable. To eliminate interference from consumers’ original travel experiences and frequency of staying in luxury hotels, we included consumers’ travel frequency and luxury brand frequency as control variables. The results showed a significant main effect of bragging language style [*F*(1,195) = 90.574, *p* < 0.001, η^2^ = 0.317], a significant main effect of similarity [*F*(1,195) = 15.942, *p* < 0.001, η^2^ = 0.076], and a significant interaction effect between bragging language and similarity [*F*(1,195) = 41.593, *p* < 0.001, η^2^ = 0.176]. As shown in Figure 4, compared with the participants in the low similarity condition (*M*_straightforward bragging_ = 5.03, SD = 0.76; *M*_humblebragging_ = 4.63, SD = 0.90; *p* = 0.008), the humblebragging language style had a stronger negative effect on brand attitude for the participants in the high similarity condition (*M*_straightforward bragging_ = 5.25, SD = 0.47; *M*_humblebragging_ = 3.45, SD = 0.87; *p <* 0.001).

#### 5.3.3. Moderated mediation analysis

We conducted a moderated mediation analysis using the PROCESS Model 7 and a bootstrap sample of 10,000. First, the results showed that similarity significantly moderated the parallel mediating effects of malicious envy (*β* = 0.132, 95% CI: [0.023, 0.298]) and trustworthiness (*β* = 0.133, 95% CI: [0.007, 0.324]). Specifically, in the low similarity condition, neither malicious envy (*β* = 0.055, 95% CI: [−0.008, 0.129]) nor trustworthiness (*β* = 0.035, 95% CI: [−0.049, 0.140]) had a mediating effect. However, in the high similarity condition, both malicious envy (*β* = 0.187, 95% CI: [0.023, 0.298]) and trustworthiness (*β* = 0.0168, 95% CI: [0.040, 0.356]) had strong mediating effects. H3 was thus supported. Table 5 shows the detailed results of the moderated mediation analysis. Table 6 shows the results with only malicious envy as a mediator, and Table 7 shows the results with only trustworthiness.

**Table 5:** Moderated mediation analysis in Study 2.

		β	SE	[LLCI, ULCI]
Direct effect		0.857	0.120	[0.620, 1.094]
Indirect effect of malicious envy	Moderated mediation	0.132	0.069	[0.023, 0.298]
	Low similarity	0.055	0.035	[−0.008, 0.129]
	High similarity	0.187	0.073	[0.023, 0.298]
Indirect effect of trustworthiness	Moderated mediation	0.133	0.083	[0.007, 0.324]
	Low similarity	0.035	0.047	[−0.049, 0.140]
	High similarity	0.168	0.081	[0.040, 0.356]

**Table 6:** Moderated mediation analysis (only malicious envy) in Study 2.

		β	SE	[LLCI, ULCI]
Direct effect		0.908	0.123	[0.666, 1.150]
Indirect effect of malicious envy	Moderated mediation	0.185	0.081	[0.050, 0.369]
	Low similarity	0.077	0.045	[−0.009, 0.169]
	High similarity	0.262	0.074	[0.130, 0.419]

**Table 7:** Moderated mediation analysis (only trustworthiness) in Study 2.

		β	SE	[LLCI, ULCI]
Direct effect		0.944	0.119	[0.709, 1.180]
Indirect effect of trustworthiness	Moderated mediation	0.174	0.096	[0.017, 0.385]
	Low similarity	0.045	0.057	[−0.064, 0.163]
	High similarity	0.220	0.085	[0.080, 0.410]

## 6. Study 3: The moderating effect of influencer expertise

### 6.1. Purpose and participants

The purpose of Study 3 was to examine the moderating effect of influencer expertise. Study 3 was a 2 (bragging type: straightforward bragging vs. humblebragging) × 2 (expertise: high vs. low) between-subjects experiment. The professional survey platform from Study 1A was used to recruit 209 participants, and the participants were randomly assigned to one of the four scenarios. Among the participants, 34.0% were men, 35.9% were between 26 and 30 years old, 75.1% were enterprise employees, 29.7% had an annual income between RMB60,000 and RMB100,000, and 43.5% stayed in luxury hotels once or twice a year.

### 6.2. Design and procedures

#### 6.2.1. Procedure

The participants were asked to imagine that they were browsing travel blogs shared by influencers on the social media site Weibo and that they saw the influencer “Ada” share his experiences and pictures from a stay at a Westin Resort hotel. Similar to Study 1, we manipulated the influencer’s bragging language style *via* WOM content, as shown in Supplementry Appendix 1. The manipulation of influencer expertise was adapted from Ki and Kim (2019). In the high expertise condition, the influencer was described as a travel expert who became popular through his travel posts. In the low expertise condition, the influencer was described as a sports blogger who gained fame through his fitness posts.

#### 6.2.2. Measures

After reading the influencer’s post, the same scales as in Study 1 were used to measure the participants’ perceptions of bragging language style, malicious envy (Cronbach’s *a =* 0.704), trustworthiness (Cronbach’s *a =* 0.822), and brand attitude (Cronbach’s *a =* 0.745). In addition, the participants’ perceptions of influencer expertise were measured with two items rated on a 7-point bipolar scale adapted from Ki and Kim (2019): “He is knowledgeable about hotels” and “He is an expert in the tourism field” (Cronbach’s *a =* 0.723). To eliminate the effect of influencer likeability, we measured likeability with one item, “I like this influencer,” rated on a 7-point bipolar scale (Chen et al., 2020).

### 6.3. Results

#### 6.3.1. Manipulation check

The participants in the humblebragging condition had stronger perceptions of humblebragging [*M*_humblebragging_ = 4.90, SD = 1.37; *M*_straightforward bragging_ = 3.87, SD = 1.49; *F*(1,207) = 26.816, *p* < 0.001] than the participants in the straightforward bragging condition, who showed stronger perceptions of straightforward boasting [*M*_humblebragging_ = 3.47, SD = 1.10; *M*_straightforward bragging_ = 5.12, SD = 1.13; *F*(1,207) = 114.460, *p* < 0.001]. In the expertise manipulation check, relative to the participants in the low expertise condition, those in the high expertise condition perceived the influencer as having a higher level of expertise [*M*_high expertise_ = 4.73, SD = 1.13; *M*_low expertise_ = 4.27, SD = 1.21; *F*(1,207) = 8.190, *p* = 0.005].

#### 6.3.2. Brand attitude

**Figure 5:**
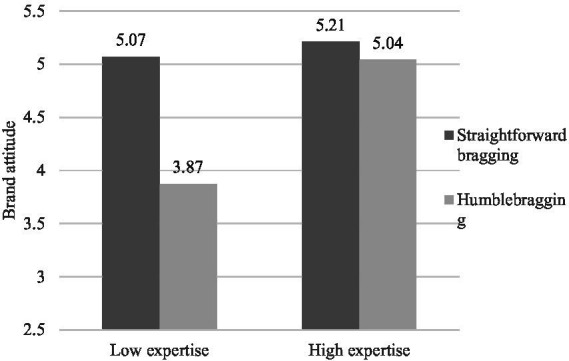
Study 3: The effect of bragging style and expertise on brand attitude.

Taking influencer likeability as a control variable, we conducted a two-way ANOVA on brand attitude. The results showed that the main effects of bragging language style [*F*(1,204) = 28.120, *p* < 0.001, η^2^ = 0.133] and influencer expertise [*F*(1,204) = 28.028, *p* < 0.001, η^2^ = 0.123] and the interaction of bragging style and expertise [*F*(1,204) = 19.035, *p* < 0.001, η^2^ = 0.079] were all significant. As shown in Figure 5, in the high expertise condition, there was no significant difference in brand attitude between the participants in the straightforward bragging and humblebragging conditions (*M*_straightforward bragging_ = 5.21, SD = 0.75; *M*_humblebragging_ = 5.04, SD = 0.80; *p* = 0.295). However, in the low expertise condition, the participants in the humblebragging condition had more negative brand attitudes (*M*_straightforward bragging_ = 5.07, SD = 1.01; *M*_humblebragging_ = 3.87, SD = 0.97; *p* < 0.001).

#### 6.3.3. Mediation analysis

We used the PROCESS Model 7 and a bootstrap sample of 10,000 to test the moderated mediation effect. The results showed that influencer expertise moderated the parallel mediating effects of malicious envy (*β* = −0.120, 95% CI: [−0.281, −0.016]) and trustworthiness (*β* = −0.319, 95% CI: [−0.567, −0.124]). As for the mediating effect of malicious envy, the indirect effect of malicious envy in the low expertise condition was stronger (*β* = 0.190, 95% CI: [0.040, 0.370]) than that in the high expertise condition (*β* = 0.070, 95% CI: [0.003, 0.167]). As for trustworthiness, the indirect effect of trustworthiness was not significant in the high expertise condition (*β* = −0.019, 95% CI: [−0.131, 0.087]), but there was a significant indirect effect in the low expertise condition (*β* = 0.300, 95% CI: [0.132, 0.509]). Thus, H4 was supported. The details are shown in Table 8. In addition, Table 9 provides the mediation results with only malicious envy, and Table 10 shows the results with only trustworthiness.

**Table 8:** Moderated mediation analysis in Study 3.

		β	SE	[LLCI, ULCI]
Direct effect		0.407	0.134	[0.143, 0.672]
Indirect effect of malicious envy	Moderated mediation	−0.120	0.070	[−0.281, −0.016]
	Low expertise	0.190	0.084	[0.040, 0.370]
	High expertise	0.070	0.041	[0.003, 0.167]
Indirect effect of trustworthiness	Moderated mediation	−0.319	0.113	[−0.567, −0.124]
	Low expertise	0.300	0.096	[0.132, 0.509]
	High expertise	−0.019	0.055	[−0.131, 0.087]

**Table 9:** Moderated mediation analysis (only malicious envy) in Study 3.

		β	SE	[LLCI, ULCI]
Direct effect		0.472	0.140	[0.196, 0.749]
Indirect effect of malicious envy	Moderated mediation	−0.189	0.086	[−0.377, −0.048]
	Low expertise	0.299	0.088	[0.141, 0.484]
	High expertise	0.110	0.056	[0.011, 0.230]

**Table 10:** Moderated mediation analysis (only trustworthiness) in Study 3.

		β	SE	[LLCI, ULCI]
Direct effect		0.516	0.128	[0.263, 0.768]
Indirect effect of trustworthiness	Moderated mediation	−0.368	0.118	[−0.621, −0.162]
	Low expertise	0.346	0.098	[0.173, 0.558]
	High expertise	−0.022	0.061	[−0.146, 0.097]

## 7. Conclusion and discussion

### 7.1. Conclusion

On social media, many luxury brands use influencers’ bragging WOM as a marketing strategy to improve consumers’ attitudes toward the luxury brands. As modesty is a virtue in the Chinese context, these influencers tend to adopt a humblebragging, rather than straightforward bragging, language style. Is humblebragging more effective than straightforward bragging? We tested the reverse effect of humblebragging in three behavioral experiments. The results of Studies 1A and 1B showed that humblebragging by influencers is more likely than straightforward bragging to trigger negative attitudes toward the mentioned luxury brands. Moreover, malicious envy and trustworthiness are dual mediators in this effect. Studies 2 and 3 explored the moderating effects of influencers’ characteristics, specifically, influencer expertise and perceived similarity. The results showed that when influencers have a low level of expertise or are perceived as highly similar to their audience, the negative effect of humblebragging on brand attitude is stronger.

### 7.2. Theoretical contributions

This research makes several theoretical contributions. First, in contrast to the studies that have highlighted the merits of humblebragging, the results of this research demonstrate the negative effect of influencers’ humblebragging on consumers’ brand attitudes. Research on WOM language style has mostly been in the context of celebrity endorsements and has highlighted the positive effects of humblebragging in advertising (Paramita and Septianto, 2021). As humility is more in line with the ethics of Chinese culture, humblebragging is widely used in social media marketing in the Chinese context. However, our results show that humblebragging (vs. straightforward bragging) on social media can easily yield negative effects and trigger negative emotions in consumers. These negative emotions can spill over to the luxury brands recommended by influencers. This research introduces bragging language style as a factor in the effectiveness of influencer marketing and discusses the potential drawbacks of marketing luxury brands with humblebragging.

Second, our findings reveal the dual psychological mechanism of social emotion and cognition underlying the negative effects of humblebragging. Most of the studies in this area have adopted the information communication perspective and have considered influencers information sources to examine the effect of humblebragging on tourism decision-making (Jang et al., 2021; Pop et al., 2022). From the perspective of the social relationship between consumers and influencers, we use social comparison theory to explain the impacts of bragging language style on brand attitudes. We argue that because of the grassroots nature of influencers, consumers regard them not only as an information source but also as targets of social comparison, which can trigger social emotions such as envy. The results of our three studies consistently show that social cognition and emotion jointly shape consumers’ brand attitudes and mediate the negative effects of humblebragging. The dual mediation mechanism we propose provides a new theoretical perspective for insight into the impact of social media influencers on brand attitudes.

Finally, this research demonstrates the interaction effect between influencers’ characteristics and their WOM content. Research on influencers has focused on the effects of influencers’ characteristics on marketing effectiveness (Lou and Yuan, 2019; Ki et al., 2020). However, insights into the potential effects of WOM content, such as language style, have been lacking. We propose that consumers’ emotional responses to humblebragging by influencers differs with the influencer’s perceived similarity and level of expertise. Although research has indicated that influencers with high similarity to consumers can more effectively promote an endorsed brand (Sánchez-Fernández and Jiménez-Castillo, 2021), we show that similarity is a double-edged sword that can aggravate the negative effects of humblebragging. This research introduces language style as a new independent variable, enriching and expanding research on influencer marketing theory.

### 7.3. Managerial implications

Although humblebragging is widely used in influencer marketing, we show that it can negatively affect a brand. In practice, humblebragging is characterized by humor and affinity and easily attracts audience attention on social media. Some managers intuitively consider humblebragging a favorable marketing tool: humblebragging seems to conform to social norms and can enhance luxury brand awareness on social media. However, our results show that humblebragging can backfire. Emotionally, humblebragging can easily trigger consumers’ malicious envy, and cognitively, it can reduce consumers’ trust in luxury brands. Moreover, humblebragging WOM can spread rapidly and potentially hurt a brand’s image, producing a negative marketing effect. Thus, luxury brand managers should exercise caution regarding humblebragging WOM when recruiting influencers for WOM marketing.

Moreover, in social media marketing, many managers tend to select influencers with high similarity to their target consumers. However, we suggest that choosing influencers similar to the target consumers can aggravate the negative effect of humblebragging WOM. In advertising, similarity between endorsers and consumers plays a positive role and helps improve the marketing effect. However, on social media, WOM content is more personalized, and influencers may adopt a bragging language style to improve their personal image while recommending luxury brands. We show that when using bragging WOM, influencers with high similarity to the target consumers may induce malicious envy and diminish luxury brand attitudes. Therefore, when brand managers choose influencers with high similarity as endorsers, they should carefully evaluate the possibility of WOM content inducing negative emotions to avoid undermining their marketing investment.

Finally, some managers choose influencers with high influence but without expertise in the product category of the brands they endorse. However, this research shows that a lack of expertise can worsen the negative effects of humblebragging. When choosing an influencer, managers often pay attention to indicators such as the influencer’s number of fans but ignore the potential impact of the influencer’s expertise. Some managers even consider influencers with little expertise more approachable and thus more effective in attracting consumers. This research shows that low expertise influencers not only fail to improve a brand’s image but can also hurt a luxury brand’s image. We suggest that influencers with high (vs. low) expertise are better able to enhance a luxury brand’s image.

### 7.4. Limitations and future research

This research has the following limitations. First, the stimuli were luxury hotels. Scholars could verify our findings with stimuli from other product and service categories, such as luxury cars. Moreover, as this research focused on the moderating effects of influencer expertise and similarity with consumers, scholars could examine how other influencer characteristics, such as perceived authenticity, moderate the effects of bragging language style. Third, we mainly focused on the joint effect of influencer characteristics and bragging language style, without considering the moderating role of brand characteristics. Future research could explore the interaction between brand characteristics and influencer language style, the relationship between tourist characteristics and brand, and the fit between influencer and brand. In addition, this research did not examine how the relative effectiveness of straightforward and humblebragging varied among consumers with different characteristics. Future research could consider the moderating effects of consumers’ characteristics, such as social comparison tendency and social relationship orientation.

## Data availability statement

The raw data supporting the conclusions of this article will be made available by the authors, without undue reservation.

## Ethics statement

The studies involving human participants were reviewed and approved by School of Management, Hainan University. The patients/participants provided their written informed consent to participate in this study.

## Author contributions

WF: conceptualization, methodology, writing—original draft preparation, investigation, project administration, supervision, and funding acquisition. DC: formal analysis, visualization, methodology, investigation, and writing—original draft preparation. HS: conceptualization, methodology, writing—original draft preparation, writing—review and editing, and funding acquisition. All authors contributed to the article and approved the submitted version.

## Funding

This work was supported by grants from the Natural Science Foundation of Hainan Province (No. 622RC625), the National Natural Science Foundation of China (NSFC) (71902120 and 72062013), and the Hainan University Research Start-up Fund (kyqd(sk)1931).

## Conflict of interest

The authors declare that the research was conducted in the absence of any commercial or financial relationships that could be construed as a potential conflict of interest.

## Publisher’s note

All claims expressed in this article are solely those of the authors and do not necessarily represent those of their affiliated organizations, or those of the publisher, the editors and the reviewers. Any product that may be evaluated in this article, or claim that may be made by its manufacturer, is not guaranteed or endorsed by the publisher.
