# Supplementary material for: The impact of social media influencers’ bragging language styles on consumers’ attitudes toward luxury brands: The dual mediation of envy and trustworthiness
Source: Front Psychol. 2023 Jan 19;13:1113655. doi: 10.3389/fpsyg.2022.1113655 (PMC9893890; doi:10.3389/fpsyg.2022.1113655)
Supplement: Supplementary file 1 [file Data_Sheet_1.docx]

Supplementary Material

# Appendix 1

## Stimuli used in study 1A

| In your spare time, you like to browse the tourist notes and pictures shared by “tourist influencer” in Little Red Book.  While browsing, you see a note posted by a tourist influencer “LEE”. According to LEE’s personal homepage, he/she has more than 121,000 followers, likes and collections of 36,000 on Little Red Book. | |
| --- | --- |
| Straightforward bragging condition | humble bragging condition |
| You can see the picture LEE recently shared about staying at the luxury Peninsula Paris. He / she has the following description of staying at the Peninsula hotel:  “After a long flight, I hoped to have a good rest in the Peninsula Paris. When I entered the room, there were complimentary champagne and a chocolate on the table. The champagne and chocolate were exclusive to VIP guests like me. In the evening, I went to the Michelin-starred restaurant downstairs. The restaurant manager recognized me and asked me many times how the food was during the meal. Generally speaking, the luxury service of the hotel is good. I recommend Peninsula Hotel to my friends who go to Paris.” | You can see the picture LEE recently shared about staying at the luxury Peninsula Paris. He / she has the following description of staying at the Peninsula hotel:  “After a long flight, I hoped to have a good rest in the Peninsula Paris. When I entered the room, there were champagne and chocolate on the table. I am trying to lose weight and I dared not eat these! In the evening, I went to the Michelin-starred restaurant downstairs. The restaurant manager recognized me and asked me many times how the food was during the meal. I felt a little annoyed since I was frequently disturbed. Generally speaking, the luxury service of the hotel is good. I recommend Peninsula Hotel to my friends who go to Paris.” |

## Stimuli used in study 1B

| In your spare time, you like to browse the travel notes and pictures shared by “influencers” on the travel social platform Weibo.  While browsing, you see a note posted by Xiao Ke, a travel influencer. According to Xiao Ke’s personal page, he or she has more than 1,531,000 followers and 343,000 comments and likes on Weibo. | |
| --- | --- |
| Straightforward bragging condition | humble bragging condition |
| You can see the travel notes and pictures of staying at the luxury Banyan Tree Shanghai On The Bund recently shared by Xiao Ke. He / she described the hotel as:  “After a long day in ‘Magic City’ Shanghai, I was a little tired and stayed in the Bund Riverview Suite at Banyan Tree Hotel. The vast river view, the huge room, how happy it was to live in it!  Before leaving, the manager sent me a white gift box printed with ‘Dessert Noble - French Raspberry macaron’. The gift box was very nice and looked very stylish!  Generally speaking, the luxurious service of the hotel is good. Promise me that you must stay at Banyan Tree Hotel when you go to Shanghai.” | You can see the travel notes and pictures of staying at the luxury Banyan Tree Shanghai On The Bund recently shared by Xiao Ke. He / she described the hotel as:  “After a long day in ‘Magic City’ Shanghai, I was a little tired and stayed in the Bund Riverview Suite at Banyan Tree Hotel. The vast river view, the huge room, how lonely it was to live in it!  Before leaving, the manager sent me a white gift box printed with ‘Dessert Noble - French Raspberry macaron’. The gift box was too crude. How can I let him know I don’t like the design?  Generally speaking, the luxurious service of the hotel is good. Promise me that you must stay at Banyan Tree Hotel when you go to Shanghai.” |

## Stimuli used in study 2

| Straightforward bragging condition | |
| --- | --- |
| High-similarity condition | Low-similarity condition |
| In your spare time, you like to browse travel notes and photos shared by tourist influencers on the travel social networking site mafengwo.com.  When you are browsing, you see a travel notes posted by a travel influencer “Dou Dou”. According to Dou Dou’s personal homepage, he is about the same age as you, just 20 years old, and he is also an ordinary college student. Recently, he became popular in a short time with a travel note. He has attracted more than 123,000 followers in the community and has become an “influencer” of the mafengwo community, reportedly earning hundreds of thousands of yuan every year.  You can see that Dou Dou recently shared his travel notes during the National Day holiday in Sanya. He stayed in the five-star Marriott Resort Hotel: “I hoped to have a good rest in the luxurious Marriott Hotel during the National Day holiday. I have stayed there several times before. The hotel provided luxurious shuttle services to VIP clients, picked me up from airport with a BMW car. Upon entering the room, there was a large basket of tropical fruit on the table, which was not for every guest. In the evening, I went to the restaurant opened by the star chef downstairs. The manager of the restaurant recognized me and warmly entertained me. During the meal, he asked me many times how the food was. Overall, the hotel service is good.” | In your spare time, you like to browse travel notes and photos shared by tourist influencers on the travel social networking site mafengwo.com.  When you are browsing, you see a travel notes posted by a travel influencer “Dou Dou”. According to Dou Dou’s personal homepage, he is a middle-aged man in his 50s and previously worked as an executive in a well-known foreign company. Recently, he became popular in a short time with a travel note. He has attracted more than 123,000 followers in the community and has become an “influencer” of the mafengwo community, reportedly earning hundreds of thousands of yuan every year.  You can see that Dou Dou recently shared his travel notes during the National Day holiday in Sanya. He stayed in the five-star Marriott Resort Hotel: “I hoped to have a good rest in the luxurious Marriott Hotel during the National Day holiday. I have stayed there several times before. The hotel provided luxurious shuttle services to VIP clients, picked me up from airport with a BMW car. Upon entering the room, there was a large basket of tropical fruit on the table, which was not for every guest. In the evening, I went to the restaurant opened by the star chef downstairs. The manager of the restaurant recognized me and warmly entertained me. During the meal, he asked me many times how the food was. Overall, the hotel service is good.” |

| humble bragging condition | |
| --- | --- |
| High-similarity condition | Low-similarity condition |
| In your spare time, you like to browse travel notes and photos shared by tourist influencers on the travel social networking site mafengwo.com.  When you are browsing, you see a travel notes posted by a travel influencer “Dou Dou”. According to Dou Dou’s personal homepage, he is about the same age as you, just 20 years old, and he is also an ordinary college student. Recently, he became popular in a short time with a travel note. He has attracted more than 123,000 followers in the community and has become an “influencer” of the mafengwo community, reportedly earning hundreds of thousands of yuan every year.  You can see that Dou Dou recently shared his travel notes during the National Day holiday in Sanya. He stayed in the five-star Marriott Resort Hotel: “I hoped to have a good rest in the luxurious Marriott Hotel during the National Day holiday. I have stayed there several times before. The hotel picked me up from airport with a BMW car, yet I felt a little carsick and uncomfortable. Upon entering the room, there was a large basket of tropical fruit on the table. I was trying to lose weight and dared not eat something with such high sugar. In the evening, I went to the restaurant opened by the star chef downstairs. The manager of the restaurant recognized me and warmly entertained me. During the meal, he asked me many times how the food was, and I felt a little annoyed when I was always disturbed. Overall, the hotel service is good.” | In your spare time, you like to browse travel notes and photos shared by tourist influencers on the travel social networking site mafengwo.com.  When you are browsing, you see a travel notes posted by a travel influencer “Dou Dou”. According to Dou Dou’s personal homepage, he is a middle-aged man in his 50s and previously worked as an executive in a well-known foreign company.Recently, he became popular in a short time with a travel note. He has attracted more than 123,000 followers in the community and has become an “influencer” of the mafengwo community, reportedly earning hundreds of thousands of yuan every year.  You can see that Dou Dou recently shared his travel notes during the National Day holiday in Sanya. He stayed in the five-star Marriott Resort Hotel: “I hoped to have a good rest in the luxurious Marriott Hotel during the National Day holiday. I have stayed there several times before. The hotel picked me up from airport with a BMW car, yet I felt a little carsick and uncomfortable. Upon entering the room, there was a large basket of tropical fruit on the table. I was trying to lose weight and dared not eat something with such high sugar. In the evening, I went to the restaurant opened by the star chef downstairs. The manager of the restaurant recognized me and warmly entertained me. During the meal, he asked me many times how the food was, and I felt a little annoyed when I was always disturbed. Overall, the hotel service is good.” |

## Stimuli used in study 3

| Straightforward bragging condition | |
| --- | --- |
| High-expertise condition | Low-expertise condition |
| In your spare time, you like to browse various micro blogs shared by influencers on Weibo.  When you browse, you see a travel blog posted by influencer “ADA”. ADA is a professional travel expert. According to her homepage, ADA is a travel expert, and become popular with a travel post in a short time. Her travel micro blogs have been read over100,000 times and the videos have been viewed 254 million times, attracting 3.331 million followers.  You can see that ADA recently shared her blog about her National Day holiday, and she stayed in Westin Resort Hotel: “I hope to have a good rest in the presidential suite in Westin, and I have stayed there several times before. I booked a hotel BMW to pick me up at the airport. When I entered the room, there was a big basket of tropical fruits on the table, which was not for every guest. In the evening, I went to the restaurant opened by the star chef downstairs for dinner. The restaurant manager remembered me and gave me a warm reception. He asked me how the food was many times during the meal. Generally speaking, the hotel service is very good.” | In your spare time, you like to browse various micro blogs shared by influencers on Weibo.  When you browse, you see a travel blog posted by influencer “ADA”. ADA is not a professional travel expert, but an influencer in fitness field. According to her homepage, ADA is a sports blogger, and become popular with a fitness post in a short time. Her fitness micro blogs have been read over 100,000 times and her videos have been viewed 254 million times, attracting 3.331 million followers.  You can see that ADA recently shared her blog about her National Day holiday, and she stayed in Westin Resort Hotel: “I hope to have a good rest in the presidential suite in Westin, and I have stayed there several times before. I booked a hotel BMW to pick me up at the airport. When I entered the room, there was a big basket of tropical fruits on the table, which was not for every guest. In the evening, I went to the restaurant opened by the star chef downstairs for dinner. The restaurant manager remembered me and gave me a warm reception. He asked me how the food was many times during the meal. Generally speaking, the hotel service is very good.” |

| humble bragging condition | |
| --- | --- |
| High-expertise condition | Low-expertise condition |
| In your spare time, you like to browse various micro blogs shared by influencers on Weibo.  When you browse, you see a travel blog posted by influencer “ADA”. ADA is a professional travel expert. According to her homepage, ADA is a travel expert, and become popular with a travel post in a short time. Her travel micro blogs have been read over100,000 times and the videos have been viewed 254 million times, attracting 3.331 million followers.  You can see that ADA recently shared her blog about her National Day holiday, and she stayed in Westin Resort Hotel: “I hope to have a good rest in the presidential suite in Westin, and I have stayed there several times before. I booked a hotel BMW to pick me up at the airport, I felt uncomfortable and got carsick. When I entered the room, there was a big basket of tropical fruits on the table. I was losing weight, so I didn’t dare to eat anything with such high sugar. In the evening, I went to the restaurant opened by the star chef downstairs for dinner. The restaurant manager remembered me and gave me a warm reception. He asked me how the food was many times during the meal, and I felt a little annoyed by being disturbed all the time. Generally speaking, the hotel service is very good.” | In your spare time, you like to browse various micro blogs shared by influencers on Weibo.  When you browse, you see a travel blog posted by influencer “ADA”. ADA is not a professional travel expert, but an influencer in fitness field. According to her homepage, ADA is a sports blogger, and become popular with a fitness post in a short time. Her fitness micro blogs have been read over 100,000 times and her videos have been viewed 254 million times, attracting 3.331 million followers.  You can see that ADA recently shared her blog about her National Day holiday, and she stayed in Westin Resort Hotel: “I hope to have a good rest in the presidential suite in Westin, and I have stayed there several times before. I booked a hotel BMW to pick me up at the airport, I felt uncomfortable and got carsick. When I entered the room, there was a big basket of tropical fruits on the table. I was losing weight, so I didn’t dare to eat anything with such high sugar. In the evening, I went to the restaurant opened by the star chef downstairs for dinner. The restaurant manager remembered me and gave me a warm reception. He asked me how the food was many times during the meal, and I felt a little annoyed by being disturbed all the time. Generally speaking, the hotel service is very good.” |

# Appendix 2. Measurement

1. **Bragging language styles: Straightforward bragging vs. Humble bragging** (adapted from Sezer et al., 2018) [ Study 1A / Study1B / Study 2 / Study 3 ]

For the Little Red Book / mafengwo / Weibo notes shared by the influencer,

1. I think his bragging is direct
2. I think his bragging contains a little complaint
3. **Influencer similarity** (adapted from Lin, 2018) [ Study 2 ]

For the influencer who shares his travel notes in mafengwo, you think:：

1. He is about the same age as me
2. He is similar to me
3. **Influencer expertise** (adapted from Ki, 2019) [ Study 3 ]

For the influencer ADA in Weibo, you think:

1. He is knowledgeable about hotels
2. He is an expert in the tourist field
3. **Malicious envy** (adapted from Lange, 2018) [ Study 1A / Study1B / Study 2 / Study 3 ]

Seeing the experience of staying at Peninsula Hotel / Banyan Tree Hotel / Marriott Hotel / Westin Hotel shared by this travel influencer:

1. I think he is enviable
2. I find him a bit annoying after seeing his sharing
3. I have some negative views on him
4. I wish it is me who stays at the Peninsula / Banyan Tree / Marriott / Westin Hotel, not him
5. **Influencer trustworthiness** (adapted from Lou & Yuan, 2019) [ Study 1A / Study1B / Study 2 / Study 3 ]

For the travel influencer LEE / Xiao Ke / Dou Dou / ADA, you think:

1. He is honest
2. He is sincere
3. He is reliable
4. His sharing is trustworthiness
5. **Brand attitude** (adapted from Lee, 2017) [ Study 1A / Study1B / Study 2 / Study 3 ]

For the Peninsula Hotel / Banyan Tree Hotel / Marriott Hotel / Westin Hotel shared by this influencer:

1. I like Peninsula / Banyan Tree / Marriott / Westin Hotel
2. I think Peninsula / Banyan Tree / Marriott / Westin Hotel is a good hotel
3. I have a negative view of Peninsula / Banyan Tree / Marriott / Westin Hotel [R]
4. **Social comparison perception (**adapted from Gibbons and Buunk, 1999**)** [ Study 1B ]

Think back to when you just browsed the Weibo share of this influencer Xiao Ke:

1. I will compare myself to this influencer
2. I will notice the difference between myself and this influencer
3. I will measure my achievements by comparing myself to this influencer
4. **Authenticity** (adapted from Lee et al., 2021) [ Study 2 ]

I think the influencer’s WOM is authentic

1. **Influencer likeability** (adapted from Chen et al., 2020) [ Study 3 ]

I like this influencer

1. **Demographic questions**

Luxury hotel frequency, Travel frequency, Social media frequency, Gender, Age, Occupation, Income.

All the above questions were assessed by Likert 7 scale (1 “strongly disagree”; 7 “strongly agree”).

**The social media posts are fictitious posts, including the content, image and use names, created by the authors for the purpose of manipulation in experiments. No third-party materials are involved. No permission is needed to reproduce the fictitious social media posts in the supplementary material.**
